# Aqua­(4-methyl­quinoline-κ*N*)[*N*-(2-oxidobenzyl­idene)glycinato-κ^3^ *O*,*N*,*O*′]copper(II) hemihydrate

**DOI:** 10.1107/S1600536807067852

**Published:** 2008-01-04

**Authors:** Zdeněk Trávníček, Jaromír Marek, Ján Vančo, Oľga Švajlenová

**Affiliations:** a Department of Inorganic Chemistry, Faculty of Science, Palacký University, Křížkovského 10, CZ-771 47 Olomouc, Czech Republic; b Laboratory of Functional Genomics and Proteomics, Institute of Experimental Biology, Faculty of Science, Masaryk University, Kamenice 5, CZ-625 00 Brno, Czech Republic; c Department of Chemical Drugs, Faculty of Pharmacy, Palackého 1/3, CZ-612 42 Brno, Czech Republic; d Department of Chemical Theory of Drugs, Faculty of Pharmacy, Comenius University, Kalinčiakova 8, SK-832 32 Bratislava, Slovak Republic

## Abstract

The title complex, [Cu(C_9_H_7_NO_3_)(C_10_H_9_N)(H_2_O)]·0.5H_2_O, crystallizes with two independent formula units in the asymmetric unit; the solvent mol­ecule is located on a twofold axis of symmetry. The Cu^II^ atom is coordinated by one tridentate *N*-salicylideneglycinate Schiff base ligand, one 4-methyl­quinoline ligand and one water mol­ecule, leading to a slightly distorted square-pyramidal N_2_O_3_ geometry. In the crystal structure, the mol­ecules are linked by O—H⋯O hydrogen bonds into linear chains in the [100] direction. The structure is further stabilized by inter­molecular C—H⋯O inter­actions and C⋯C contacts with C⋯C = 3.3058 (2), 3.3636 (2) and 3.3946 (2) Å.

## Related literature

For synthesis, see: Kishita *et al.* (1964 ▶). For related literature, see: Katsuki (2003 ▶); Vančo *et al.* (2004 ▶, 2008 ▶); Bauerová *et al.* (2005 ▶). For related structures, see: Valent *et al.* (2002 ▶); Warda (1998*a* ▶,*b* ▶,*c* ▶,*d* ▶).
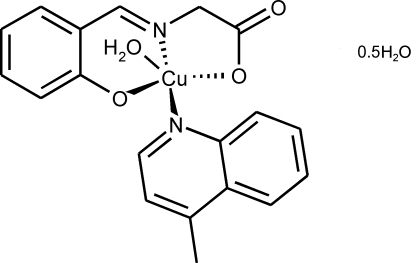


## Experimental

### 

#### Crystal data

[Cu(C_9_H_7_NO_3_)(C_10_H_9_N)(H_2_O)]·0.5H_2_O
*M* *_r_* = 410.9
Monoclinic, 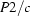

*a* = 10.0966 (7) Å
*b* = 12.3483 (6) Å
*c* = 28.8133 (17) Å
β = 97.730 (6)°
*V* = 3559.7 (4) Å^3^
*Z* = 8
Mo *K*α radiation
μ = 1.26 mm^−1^
*T* = 120 (2) K
0.30 × 0.25 × 0.25 mm

#### Data collection

Kuma KM-4-CCD diffractometer
Absorption correction: multi-scan (*CrysAlis RED*; Oxford Diffraction, 2006 ▶) *T*_min_ = 0.690, *T*_max_ = 0.729
19645 measured reflections
6242 independent reflections
4225 reflections with *I* > 2σ(*I*)
*R*_int_ = 0.049

#### Refinement

*R*[*F* ^2^ > 2σ(*F* ^2^)] = 0.047
*wR*(*F* ^2^) = 0.113
*S* = 1.09
6242 reflections
499 parameters
6 restraints
H atoms treated by a mixture of independent and constrained refinement
Δρ_max_ = 0.66 e Å^−3^
Δρ_min_ = −0.54 e Å^−3^

### 

Data collection: *CrysAlis CCD* (Oxford Diffraction, 2006 ▶); cell refinement: *CrysAlis RED* (Oxford Diffraction, 2006 ▶); data reduction: *CrysAlis RED*; program(s) used to solve structure: *SHELXS97* (Sheldrick, 1990 ▶); program(s) used to refine structure: *SHELXL97* (Sheldrick, 1997 ▶); molecular graphics: *DIAMOND* (Brandenburg, 2006 ▶); software used to prepare material for publication: *SHELXL97*.

## Supplementary Material

Crystal structure: contains datablocks I, global. DOI: 10.1107/S1600536807067852/tk2236sup1.cif

Structure factors: contains datablocks I. DOI: 10.1107/S1600536807067852/tk2236Isup2.hkl

Additional supplementary materials:  crystallographic information; 3D view; checkCIF report

## Figures and Tables

**Table 1:** Hydrogen-bond geometry (Å, °)

*D*—H⋯*A*	*D*—H	H⋯*A*	*D*⋯*A*	*D*—H⋯*A*
O4—H4*V*⋯O102^i^	0.879 (19)	1.88 (2)	2.756 (4)	174 (4)
O4—H4*W*⋯O2^ii^	0.87 (4)	2.01 (3)	2.825 (4)	155 (4)
O5—H5*V*⋯O101^iii^	0.890 (19)	1.99 (2)	2.865 (4)	169 (4)
O6—H6*V*⋯O1	0.876 (19)	2.01 (2)	2.867 (4)	165 (4)
O104—H4*Y*⋯O2	0.879 (19)	1.90 (2)	2.751 (4)	162 (4)
O104—H4*Z*⋯O102^iii^	0.87 (4)	1.98 (2)	2.823 (4)	162 (4)

Symmetry codes: (i) 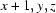; (ii) 
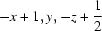
; (iii) 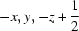.

**Fig. 1.**
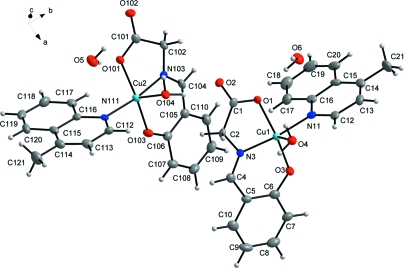
The molecular structures of the independent complex and water molecules in (I), showing 50% probability displacement ellipsoids and the atom-numbering scheme.

**Fig. 2.**
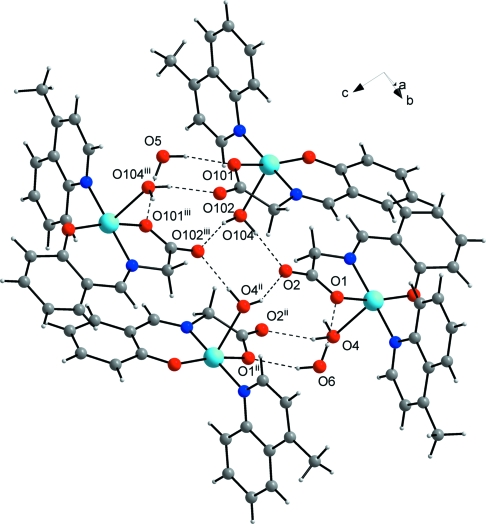
Part of the crystal structure of (I), showing the formation of O—H···O hydrogen bonds as dashed lines [Symmetry codes: (ii) -x + 1, y, -z + 1/2; (iii) -x, y, -z + 1/2].

**Fig. 3.**
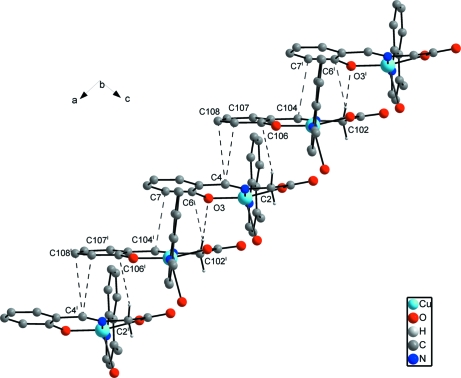
Part of the crystal structure of (I), showing the formation of intermolecular C—H···O interactions (dashed lines) and C···C contacts (dashed lines) with C102···O3i = 3.6974 (2), C2···C106 = 3.5391 (2), C4···C107 = 3.3636 (2), C4···C108 = 3.3058 (2), C104···C7i = 3.3946 (2), and C102···C6i = 3.5122 (2) Å [Symmetry code: (i) x + 1, y, z]. Water molecules of crystallization and H-atoms not involved in hydrogen bonding are omitted for clarity.

## supplementary crystallographic information

### Comment

Schiff bases, as condensation products of carbonyls and amines, and their coordination compounds find their utilization in different branches of chemical technology (Katsuki, 2003) and participate in some biochemical pathways, *e.g.* transamination processes, catalyzed by metalloenzymes.

In connection with our recent studies on copper and zinc salicylidene-derived Schiff base complexes, we report now the structure of (I). The Schiff base (Salgly) ligand represents a condensation product of salicylaldehyde and glycine. The title complex, in the form of an anhydrous compound, showed significant microbistatic and fungistatic effects (Valent *et al.*, 2002). Moreover, similar compounds derived from different *N*-salicylideneamino acids have been intensively studied and showed some remarkable biological features, from which the antioxidant (Vančo *et al.*, 2008), antiflogistic, antirheumatic (Bauerová *et al.*, 2005), or antidiabetic activities (Vančo *et al.*, 2004) could be considered as the most interesting.

To date, only four X-ray structures of monomeric copper(II) complexes involving the aqua-(*N*-salicylideneglycinato-κ*O*,*N*,*O*')copper(II) moiety in combination with another *N*-donor ligand, *i.e.* an alkylated pyridine derivative, have been reported (Warda, 1998*a*-d). While the present structure is the first one with two-ring one-N-donor aromatic ligand, *i.e.* 4-methylquinoline (Mqui), there are similarities in the interatomic parameters defining the coordination of the central atom in these complexes.

Two independent formula units of Cu(Salgly)(Mqui)(H_2_O).1/2(H_2_O) comprise the asymmetric unit of (I), see Fig. 1; each of the solvent water molecules lies on a 2-fold axis. Each Cu^II^ atom is chelated by two N atoms and a O atom, derived from the Salgly ligand, one N atom from the Mqui ligand. The resultant penta-coordinated geometry is completed by a water molecule. The O atom of the latter ligand occupies an apical position in a slightly distorted square-pyramidal coordination geometry [τ = 0.102 (for Cu1) and 0.091 (for Cu2)]. The bond distances of Cu—N_azomethine_ [1.927 (3) and 1.926 (3) Å], Cu—N_imine_ [1.993 (3) and 1.982 (3) Å], Cu—O_carboxy_ [1.986 (3) and 1.982 (3) Å], Cu—O_phenoxy_ [1.910 (3) and 1.909 (3) Å] and Cu—O_water_ [2.371 (3) and 2.367 (3) Å] as well as O_carboxy_—Cu—O_phenoxy_ [166.61 (12) and 166.81 (12) °] and N_azomethine_—Cu—N_imine_ [172.74 (14) and 172.24 (14) °] bond angles are quite similar in the independent complex molecules.

The primary intermolecular contacts in the crystal structure are of the type O—H···O (Fig. 2 & Table 1) and involve both coordinated and free water molecules, and both O atoms of carboxy groups, joining them into linear chains in the [100] direction. Moreover, the secondary structure is stabilized by intermolecular C—H···O interactions and C···C contacts (Fig. 3).

### Experimental

The title complex, (I), was prepared by the reaction of an ethanol/water solution (2:1, *v*/*v*) of aqua-(*N*-salicylideneglycinato)copper(II) hemihydrate (Kishita *et al.*, 1964) with an ethanolic solution of 4-methylquinoline in the molar ratio of 1:4. The reaction mixture was stirred at 60 °C for 30 minutes. After cooling overnight, well developed single crystals of (I) suitable for X-ray analysis were isolated.

### Refinement

C-bound H-atoms were included in the riding model approximation with C–H distances of 0.95 Å (C_aromatic_), 0.98 Å (CH_3_) and 0.99 Å (CH_2_), and with *U*_iso_(H) values of 1.2*U*_eq_(CH_2_, C_aromatic_) or 1.5*U*_eq_(C_methyl_). The O-bound H atoms were refined, with the O—H distances restrained to 0.90 (2) Å and with *U*_iso_(H) values of 1.5*U*_eq_(O_water_); distances are given in Table 1.

### Crystal data

**Table:**

[Cu(C_9_H_7_NO_3_)(C_10_H_9_N)(H_2_O)]·0.5H_2_O	*F*_000_ = 1696
*M**_r_* = 410.9	*D*_x_ = 1.533 Mg m^−^^3^
Monoclinic, *P*2/*c*	Mo *K*α radiation λ = 0.71073 Å
Hall symbol: -P 2yc	Cell parameters from 3906 reflections
*a* = 10.0966 (7) Å	θ = 2.6–26.5º
*b* = 12.3483 (6) Å	µ = 1.26 mm^−^^1^
*c* = 28.8133 (17) Å	*T* = 120 (2) K
β = 97.730 (6)º	Prism, blue
*V* = 3559.7 (4) Å^3^	0.30 × 0.25 × 0.25 mm
*Z* = 8	

### Data collection

**Table:**

Kuma KM-4-CCD diffractometer	6242 independent reflections
Radiation source: fine-focus sealed tube	4225 reflections with *I* > 2σ(*I*)
Monochromator: Enhance (Oxford Diffraction)	*R*_int_ = 0.049
Detector resolution: 8.3611 pixels mm^-1^	θ_max_ = 25.0º
*T* = 120(2) K	θ_min_ = 3.2º
rotation method ω scans	*h* = −12→11
Absorption correction: multi-scan(CrysAlis RED; Oxford Diffraction, 2006)	*k* = −14→13
*T*_min_ = 0.690, *T*_max_ = 0.729	*l* = −34→34
19645 measured reflections	

### Refinement

**Table:**

Refinement on *F*^2^	Secondary atom site location: difference Fourier map
Least-squares matrix: full	Hydrogen site location: inferred from neighbouring sites
*R*[*F*^2^ > 2σ(*F*^2^)] = 0.047	H atoms treated by a mixture of independent and constrained refinement
*wR*(*F*^2^) = 0.113	*w* = 1/[σ^2^(*F*_o_^2^) + (0.04*P*)^2^ + 2.5*P*] where *P* = (*F*_o_^2^ + 2*F*_c_^2^)/3
*S* = 1.09	(Δ/σ)_max_ = 0.001
6242 reflections	Δρ_max_ = 0.66 e Å^−^^3^
499 parameters	Δρ_min_ = −0.54 e Å^−^^3^
6 restraints	Extinction correction: none
Primary atom site location: structure-invariant direct methods	

### Special details

**Table:**

Geometry. All e.s.d.'s (except the e.s.d. in the dihedral angle between two l.s. planes) are estimated using the full covariance matrix. The cell e.s.d.'s are taken into account individually in the estimation of e.s.d.'s in distances, angles and torsion angles; correlations between e.s.d.'s in cell parameters are only used when they are defined by crystal symmetry. An approximate (isotropic) treatment of cell e.s.d.'s is used for estimating e.s.d.'s involving l.s. planes.
Refinement. Refinement of *F*^2^ against ALL reflections. The weighted *R*-factor *wR* and goodness of fit *S* are based on *F*^2^, conventional *R*-factors *R* are based on *F*, with *F* set to zero for negative *F*^2^. The threshold expression of *F*^2^ > σ(*F*^2^) is used only for calculating *R*-factors(gt) *etc*. and is not relevant to the choice of reflections for refinement. *R*-factors based on *F*^2^ are statistically about twice as large as those based on *F*, and *R*- factors based on ALL data will be even larger.

### Fractional atomic coordinates and isotropic or equivalent isotropic displacement parameters (Å^2^)

**Table:**

	*x*	*y*	*z*	*U*_iso_*/*U*_eq_	
Cu1	0.64949 (5)	0.89277 (4)	0.146484 (18)	0.01660 (15)	
Cu2	0.14540 (5)	0.52717 (4)	0.145347 (17)	0.01668 (15)	
O1	0.4892 (3)	0.8873 (2)	0.17954 (9)	0.0175 (7)	
O2	0.3605 (3)	0.7712 (2)	0.21210 (9)	0.0198 (7)	
O3	0.7735 (3)	0.8959 (2)	0.10193 (10)	0.0212 (7)	
O4	0.7828 (3)	0.8607 (2)	0.21941 (10)	0.0199 (7)	
H4V	0.802 (4)	0.7916 (18)	0.2171 (15)	0.030*	
H4W	0.733 (4)	0.854 (4)	0.2415 (12)	0.030*	
O101	−0.0123 (3)	0.5325 (2)	0.17948 (9)	0.0179 (7)	
O102	−0.1400 (3)	0.6477 (2)	0.21334 (9)	0.0208 (7)	
O103	0.2671 (3)	0.5247 (2)	0.10008 (10)	0.0221 (7)	
O104	0.2815 (3)	0.5589 (2)	0.21757 (10)	0.0195 (7)	
H4Y	0.323 (4)	0.621 (2)	0.2148 (15)	0.029*	
H4Z	0.231 (4)	0.572 (4)	0.2391 (12)	0.029*	
C1	0.4519 (4)	0.7919 (3)	0.18899 (13)	0.0168 (9)	
C2	0.5282 (4)	0.6981 (3)	0.17115 (14)	0.0170 (9)	
H2A	0.5803	0.6602	0.1979	0.020*	
H2B	0.4646	0.6458	0.1543	0.020*	
N3	0.6186 (3)	0.7393 (3)	0.13948 (11)	0.0142 (8)	
C4	0.6656 (4)	0.6760 (3)	0.11074 (14)	0.0195 (10)	
H4	0.6388	0.6022	0.1106	0.023*	
C5	0.7556 (4)	0.7073 (3)	0.07865 (13)	0.0179 (9)	
C6	0.8021 (4)	0.8155 (4)	0.07522 (14)	0.0214 (10)	
C7	0.8874 (4)	0.8365 (4)	0.04121 (15)	0.0248 (10)	
H7	0.9184	0.9082	0.0375	0.030*	
C8	0.9263 (5)	0.7556 (4)	0.01340 (16)	0.0308 (12)	
H8	0.9839	0.7725	−0.0091	0.037*	
C9	0.8833 (5)	0.6487 (4)	0.01734 (15)	0.0306 (12)	
H9	0.9121	0.5929	−0.0017	0.037*	
C10	0.7983 (4)	0.6271 (4)	0.04955 (15)	0.0264 (11)	
H10	0.7672	0.5550	0.0522	0.032*	
N11	0.6567 (3)	1.0531 (3)	0.15411 (11)	0.0175 (8)	
C12	0.7484 (4)	1.0987 (3)	0.18422 (14)	0.0184 (10)	
H12	0.8134	1.0533	0.2014	0.022*	
C13	0.7568 (4)	1.2106 (4)	0.19281 (15)	0.0237 (10)	
H13	0.8264	1.2390	0.2150	0.028*	
C14	0.6646 (4)	1.2783 (3)	0.16915 (15)	0.0225 (10)	
C15	0.5640 (4)	1.2326 (3)	0.13509 (14)	0.0200 (10)	
C16	0.5637 (4)	1.1192 (3)	0.12792 (13)	0.0179 (9)	
C17	0.4669 (4)	1.0718 (4)	0.09435 (15)	0.0258 (11)	
H17	0.4673	0.9958	0.0892	0.031*	
C18	0.3730 (5)	1.1347 (4)	0.06929 (16)	0.0321 (12)	
H18	0.3076	1.1023	0.0468	0.039*	
C19	0.3712 (5)	1.2475 (4)	0.07621 (17)	0.0336 (12)	
H19	0.3054	1.2907	0.0582	0.040*	
C20	0.4634 (4)	1.2946 (4)	0.10850 (16)	0.0272 (11)	
H20	0.4603	1.3706	0.1133	0.033*	
C21	0.6709 (5)	1.3984 (3)	0.17792 (18)	0.0366 (13)	
H21A	0.7418	1.4142	0.2037	0.055*	
H21B	0.5850	1.4235	0.1862	0.055*	
H21C	0.6900	1.4359	0.1496	0.055*	
C101	−0.0500 (4)	0.6277 (3)	0.18915 (14)	0.0173 (9)	
C102	0.0258 (4)	0.7220 (3)	0.17074 (13)	0.0175 (9)	
H10A	−0.0382	0.7751	0.1547	0.021*	
H10B	0.0806	0.7591	0.1971	0.021*	
N103	0.1120 (3)	0.6801 (3)	0.13791 (11)	0.0159 (8)	
C104	0.1625 (4)	0.7452 (3)	0.11094 (13)	0.0174 (9)	
H104	0.1374	0.8193	0.1117	0.021*	
C105	0.2556 (4)	0.7147 (3)	0.07917 (14)	0.0192 (10)	
C106	0.3004 (4)	0.6060 (3)	0.07514 (14)	0.0184 (10)	
C107	0.3904 (4)	0.5879 (4)	0.04231 (14)	0.0225 (10)	
H107	0.4198	0.5162	0.0375	0.027*	
C108	0.4363 (4)	0.6710 (4)	0.01733 (15)	0.0258 (11)	
H108	0.4982	0.6555	−0.0039	0.031*	
C109	0.3949 (4)	0.7773 (4)	0.02211 (15)	0.0260 (11)	
H109	0.4282	0.8343	0.0048	0.031*	
C110	0.3047 (4)	0.7971 (4)	0.05253 (14)	0.0233 (10)	
H110	0.2743	0.8692	0.0558	0.028*	
N111	0.1532 (3)	0.3675 (3)	0.15199 (11)	0.0135 (7)	
C112	0.2498 (4)	0.3203 (3)	0.18149 (14)	0.0186 (10)	
H112	0.3161	0.3653	0.1982	0.022*	
C113	0.2587 (4)	0.2090 (3)	0.18907 (14)	0.0212 (10)	
H113	0.3303	0.1805	0.2103	0.025*	
C114	0.1658 (5)	0.1406 (4)	0.16633 (15)	0.0227 (10)	
C115	0.0611 (4)	0.1873 (3)	0.13443 (14)	0.0201 (10)	
C116	0.0567 (4)	0.3009 (3)	0.12845 (14)	0.0184 (10)	
C117	−0.0458 (4)	0.3493 (4)	0.09754 (14)	0.0204 (10)	
H117	−0.0468	0.4256	0.0932	0.024*	
C118	−0.1436 (4)	0.2872 (4)	0.07372 (15)	0.0275 (11)	
H118	−0.2133	0.3205	0.0532	0.033*	
C119	−0.1422 (5)	0.1741 (4)	0.07931 (15)	0.0272 (11)	
H119	−0.2111	0.1313	0.0627	0.033*	
C120	−0.0422 (4)	0.1257 (4)	0.10848 (15)	0.0250 (11)	
H120	−0.0415	0.0491	0.1115	0.030*	
C121	0.1741 (5)	0.0218 (4)	0.17441 (18)	0.0360 (13)	
H12A	0.2479	0.0059	0.1992	0.054*	
H12B	0.1897	−0.0147	0.1454	0.054*	
H12C	0.0900	−0.0040	0.1840	0.054*	
O5	0.0000	0.3678 (4)	0.2500	0.0289 (11)	
H5V	0.005 (5)	0.412 (3)	0.2745 (11)	0.043*	
O6	0.5000	1.0521 (4)	0.2500	0.0267 (10)	
H6V	0.486 (5)	1.010 (3)	0.2254 (11)	0.040*	

### Atomic displacement parameters (Å^2^)

**Table:**

	*U*^11^	*U*^22^	*U*^33^	*U*^12^	*U*^13^	*U*^23^
Cu1	0.0187 (3)	0.0130 (3)	0.0189 (3)	−0.0010 (2)	0.0059 (2)	−0.0003 (2)
Cu2	0.0200 (3)	0.0126 (3)	0.0184 (3)	0.0014 (2)	0.0060 (2)	0.0005 (2)
O1	0.0172 (16)	0.0111 (15)	0.0255 (17)	−0.0003 (12)	0.0073 (13)	−0.0005 (13)
O2	0.0188 (17)	0.0211 (16)	0.0210 (16)	−0.0043 (13)	0.0081 (13)	−0.0015 (13)
O3	0.0272 (18)	0.0169 (16)	0.0217 (16)	−0.0041 (13)	0.0112 (13)	−0.0035 (13)
O4	0.0220 (17)	0.0182 (16)	0.0200 (16)	0.0005 (14)	0.0051 (13)	0.0009 (14)
O101	0.0201 (16)	0.0128 (16)	0.0217 (16)	0.0004 (12)	0.0062 (13)	−0.0003 (12)
O102	0.0224 (17)	0.0204 (16)	0.0214 (16)	0.0026 (13)	0.0099 (13)	0.0018 (13)
O103	0.0284 (18)	0.0158 (16)	0.0248 (17)	0.0030 (13)	0.0136 (14)	0.0027 (13)
O104	0.0240 (18)	0.0180 (16)	0.0177 (16)	−0.0030 (14)	0.0067 (13)	−0.0027 (14)
C1	0.013 (2)	0.021 (2)	0.014 (2)	−0.0017 (19)	−0.0035 (17)	0.0011 (18)
C2	0.016 (2)	0.018 (2)	0.017 (2)	−0.0066 (18)	0.0032 (17)	0.0014 (18)
N3	0.018 (2)	0.0127 (17)	0.0128 (18)	−0.0022 (15)	0.0054 (14)	−0.0002 (15)
C4	0.019 (2)	0.021 (2)	0.017 (2)	0.0015 (19)	−0.0022 (18)	0.0001 (19)
C5	0.017 (2)	0.022 (2)	0.014 (2)	0.0056 (19)	0.0014 (17)	−0.0001 (19)
C6	0.019 (2)	0.027 (3)	0.018 (2)	0.004 (2)	0.0001 (18)	0.000 (2)
C7	0.018 (2)	0.031 (3)	0.027 (3)	0.002 (2)	0.006 (2)	0.002 (2)
C8	0.025 (3)	0.046 (3)	0.022 (3)	0.003 (2)	0.007 (2)	0.002 (2)
C9	0.031 (3)	0.040 (3)	0.021 (2)	0.011 (2)	0.005 (2)	−0.008 (2)
C10	0.025 (3)	0.027 (3)	0.025 (2)	0.005 (2)	−0.003 (2)	−0.002 (2)
N11	0.017 (2)	0.0182 (19)	0.019 (2)	0.0015 (16)	0.0072 (16)	0.0020 (16)
C12	0.021 (2)	0.020 (2)	0.016 (2)	−0.0028 (19)	0.0087 (18)	0.0025 (19)
C13	0.025 (3)	0.023 (3)	0.024 (2)	−0.009 (2)	0.007 (2)	−0.001 (2)
C14	0.029 (3)	0.019 (2)	0.022 (2)	−0.002 (2)	0.014 (2)	0.000 (2)
C15	0.021 (2)	0.021 (2)	0.020 (2)	0.007 (2)	0.0108 (19)	0.0043 (19)
C16	0.022 (2)	0.022 (2)	0.011 (2)	0.0025 (19)	0.0076 (18)	0.0057 (18)
C17	0.024 (3)	0.028 (3)	0.026 (3)	−0.004 (2)	0.004 (2)	0.004 (2)
C18	0.029 (3)	0.043 (3)	0.023 (3)	−0.004 (2)	0.000 (2)	0.009 (2)
C19	0.027 (3)	0.043 (3)	0.032 (3)	0.008 (2)	0.008 (2)	0.019 (3)
C20	0.027 (3)	0.024 (3)	0.034 (3)	0.006 (2)	0.016 (2)	0.009 (2)
C21	0.042 (3)	0.015 (3)	0.054 (4)	0.001 (2)	0.011 (3)	−0.004 (2)
C101	0.020 (2)	0.016 (2)	0.014 (2)	0.0024 (19)	−0.0038 (18)	0.0014 (18)
C102	0.025 (2)	0.014 (2)	0.013 (2)	−0.0009 (19)	0.0046 (18)	−0.0011 (18)
N103	0.0133 (19)	0.0157 (19)	0.0182 (19)	−0.0009 (15)	0.0001 (15)	−0.0004 (16)
C104	0.021 (2)	0.012 (2)	0.018 (2)	0.0010 (18)	−0.0033 (18)	−0.0013 (18)
C105	0.017 (2)	0.022 (2)	0.017 (2)	−0.0003 (19)	−0.0017 (18)	−0.0009 (19)
C106	0.019 (2)	0.023 (2)	0.013 (2)	−0.0007 (19)	0.0009 (18)	−0.0006 (19)
C107	0.023 (3)	0.027 (3)	0.018 (2)	0.003 (2)	0.0040 (19)	−0.004 (2)
C108	0.023 (3)	0.036 (3)	0.020 (2)	−0.004 (2)	0.0061 (19)	−0.001 (2)
C109	0.028 (3)	0.030 (3)	0.021 (2)	−0.009 (2)	0.004 (2)	0.007 (2)
C110	0.030 (3)	0.019 (2)	0.021 (2)	−0.003 (2)	0.002 (2)	0.0013 (19)
N111	0.0168 (19)	0.0130 (17)	0.0121 (17)	0.0010 (15)	0.0074 (14)	−0.0007 (15)
C112	0.015 (2)	0.020 (2)	0.022 (2)	0.0020 (19)	0.0039 (18)	0.0002 (19)
C113	0.030 (3)	0.022 (2)	0.012 (2)	0.005 (2)	0.0030 (18)	0.0027 (19)
C114	0.031 (3)	0.017 (2)	0.021 (2)	0.002 (2)	0.010 (2)	0.002 (2)
C115	0.028 (3)	0.015 (2)	0.020 (2)	0.0014 (19)	0.0118 (19)	−0.0003 (19)
C116	0.018 (2)	0.017 (2)	0.022 (2)	−0.0004 (19)	0.0114 (18)	−0.0010 (19)
C117	0.024 (3)	0.021 (2)	0.017 (2)	0.002 (2)	0.0061 (19)	−0.0029 (19)
C118	0.020 (3)	0.039 (3)	0.024 (3)	−0.002 (2)	0.002 (2)	−0.001 (2)
C119	0.026 (3)	0.033 (3)	0.026 (3)	−0.011 (2)	0.013 (2)	−0.012 (2)
C120	0.032 (3)	0.019 (2)	0.027 (3)	−0.008 (2)	0.011 (2)	−0.007 (2)
C121	0.049 (3)	0.021 (3)	0.038 (3)	−0.001 (2)	0.006 (3)	0.002 (2)
O5	0.043 (3)	0.022 (2)	0.023 (3)	0.000	0.008 (2)	0.000
O6	0.040 (3)	0.024 (3)	0.017 (2)	0.000	0.005 (2)	0.000

### Geometric parameters (Å, °)

**Table:**

Cu1—O3	1.910 (3)	C17—H17	0.9500
Cu1—N3	1.927 (3)	C18—C19	1.408 (7)
Cu1—O1	1.986 (3)	C18—H18	0.9500
Cu1—N11	1.993 (3)	C19—C20	1.355 (7)
Cu1—O4	2.371 (3)	C19—H19	0.9500
Cu2—O103	1.909 (3)	C20—H20	0.9500
Cu2—N103	1.926 (3)	C21—H21A	0.9800
Cu2—N111	1.982 (3)	C21—H21B	0.9800
Cu2—O101	1.982 (3)	C21—H21C	0.9800
Cu2—O104	2.367 (3)	C101—C102	1.527 (5)
O1—C1	1.276 (5)	C102—N103	1.463 (5)
O2—C1	1.235 (5)	C102—H10A	0.9900
O3—C6	1.312 (5)	C102—H10B	0.9900
O4—H4V	0.879 (19)	N103—C104	1.272 (5)
O4—H4W	0.87 (4)	C104—C105	1.447 (6)
O101—C101	1.278 (5)	C104—H104	0.9500
O102—C101	1.242 (5)	C105—C110	1.405 (6)
O103—C106	1.305 (5)	C105—C106	1.426 (6)
O104—H4Y	0.879 (19)	C106—C107	1.415 (6)
O104—H4Z	0.87 (4)	C107—C108	1.369 (6)
C1—C2	1.518 (6)	C107—H107	0.9500
C2—N3	1.465 (5)	C108—C109	1.390 (6)
C2—H2A	0.9900	C108—H108	0.9500
C2—H2B	0.9900	C109—C110	1.368 (6)
N3—C4	1.276 (5)	C109—H109	0.9500
C4—C5	1.434 (5)	C110—H110	0.9500
C4—H4	0.9500	N111—C112	1.338 (5)
C5—C10	1.403 (6)	N111—C116	1.381 (5)
C5—C6	1.424 (6)	C112—C113	1.392 (6)
C6—C7	1.413 (6)	C112—H112	0.9500
C7—C8	1.371 (6)	C113—C114	1.363 (6)
C7—H7	0.9500	C113—H113	0.9500
C8—C9	1.398 (6)	C114—C115	1.426 (6)
C8—H8	0.9500	C114—C121	1.486 (6)
C9—C10	1.373 (6)	C115—C116	1.414 (6)
C9—H9	0.9500	C115—C120	1.419 (6)
C10—H10	0.9500	C116—C117	1.404 (6)
N11—C12	1.308 (5)	C117—C118	1.361 (6)
N11—C16	1.388 (5)	C117—H117	0.9500
C12—C13	1.404 (6)	C118—C119	1.406 (6)
C12—H12	0.9500	C118—H118	0.9500
C13—C14	1.364 (6)	C119—C120	1.362 (6)
C13—H13	0.9500	C119—H119	0.9500
C14—C15	1.430 (6)	C120—H120	0.9500
C14—C21	1.505 (6)	C121—H12A	0.9800
C15—C20	1.413 (6)	C121—H12B	0.9800
C15—C16	1.416 (6)	C121—H12C	0.9800
C16—C17	1.407 (6)	O5—H5V	0.890 (19)
C17—C18	1.357 (6)	O6—H6V	0.876 (19)
			
O3—Cu1—N3	93.49 (12)	C17—C18—C19	120.8 (4)
O3—Cu1—O1	166.61 (12)	C17—C18—H18	119.6
N3—Cu1—O1	83.42 (12)	C19—C18—H18	119.6
O3—Cu1—N11	92.10 (12)	C20—C19—C18	120.1 (4)
N3—Cu1—N11	172.74 (14)	C20—C19—H19	120.0
O1—Cu1—N11	90.08 (12)	C18—C19—H19	120.0
O3—Cu1—O4	104.68 (11)	C19—C20—C15	121.2 (4)
N3—Cu1—O4	89.61 (12)	C19—C20—H20	119.4
O1—Cu1—O4	88.37 (11)	C15—C20—H20	119.4
N11—Cu1—O4	93.45 (12)	C14—C21—H21A	109.5
O103—Cu2—N103	93.38 (13)	C14—C21—H21B	109.5
O103—Cu2—N111	91.68 (12)	H21A—C21—H21B	109.5
N103—Cu2—N111	172.24 (14)	C14—C21—H21C	109.5
O103—Cu2—O101	166.81 (12)	H21A—C21—H21C	109.5
N103—Cu2—O101	83.24 (12)	H21B—C21—H21C	109.5
N111—Cu2—O101	90.51 (12)	O102—C101—O101	124.6 (4)
O103—Cu2—O104	104.63 (11)	O102—C101—C102	118.8 (4)
N103—Cu2—O104	90.45 (12)	O101—C101—C102	116.6 (4)
N111—Cu2—O104	93.96 (12)	N103—C102—C101	108.9 (3)
O101—Cu2—O104	88.19 (11)	N103—C102—H10A	109.9
C1—O1—Cu1	114.6 (3)	C101—C102—H10A	109.9
C6—O3—Cu1	126.5 (3)	N103—C102—H10B	109.9
Cu1—O4—H4V	101 (3)	C101—C102—H10B	109.9
Cu1—O4—H4W	110 (3)	H10A—C102—H10B	108.3
H4V—O4—H4W	96 (4)	C104—N103—C102	119.6 (3)
C101—O101—Cu2	114.9 (3)	C104—N103—Cu2	127.5 (3)
C106—O103—Cu2	126.9 (3)	C102—N103—Cu2	112.7 (2)
Cu2—O104—H4Y	107 (3)	N103—C104—C105	124.8 (4)
Cu2—O104—H4Z	110 (3)	N103—C104—H104	117.6
H4Y—O104—H4Z	103 (4)	C105—C104—H104	117.6
O2—C1—O1	124.7 (4)	C110—C105—C106	119.9 (4)
O2—C1—C2	118.3 (4)	C110—C105—C104	117.7 (4)
O1—C1—C2	117.0 (3)	C106—C105—C104	122.4 (4)
N3—C2—C1	109.4 (3)	O103—C106—C107	118.8 (4)
N3—C2—H2A	109.8	O103—C106—C105	124.9 (4)
C1—C2—H2A	109.8	C107—C106—C105	116.3 (4)
N3—C2—H2B	109.8	C108—C107—C106	121.7 (4)
C1—C2—H2B	109.8	C108—C107—H107	119.1
H2A—C2—H2B	108.2	C106—C107—H107	119.1
C4—N3—C2	120.6 (3)	C107—C108—C109	121.9 (4)
C4—N3—Cu1	126.9 (3)	C107—C108—H108	119.1
C2—N3—Cu1	112.4 (2)	C109—C108—H108	119.1
N3—C4—C5	125.2 (4)	C110—C109—C108	117.9 (4)
N3—C4—H4	117.4	C110—C109—H109	121.1
C5—C4—H4	117.4	C108—C109—H109	121.1
C10—C5—C6	119.4 (4)	C109—C110—C105	122.3 (4)
C10—C5—C4	117.9 (4)	C109—C110—H110	118.8
C6—C5—C4	122.8 (4)	C105—C110—H110	118.8
O3—C6—C7	118.1 (4)	C112—N111—C116	117.3 (3)
O3—C6—C5	124.6 (4)	C112—N111—Cu2	120.7 (3)
C7—C6—C5	117.3 (4)	C116—N111—Cu2	121.9 (3)
C8—C7—C6	121.4 (4)	N111—C112—C113	123.7 (4)
C8—C7—H7	119.3	N111—C112—H112	118.1
C6—C7—H7	119.3	C113—C112—H112	118.1
C7—C8—C9	121.6 (4)	C114—C113—C112	120.7 (4)
C7—C8—H8	119.2	C114—C113—H113	119.6
C9—C8—H8	119.2	C112—C113—H113	119.6
C10—C9—C8	117.9 (4)	C113—C114—C115	117.5 (4)
C10—C9—H9	121.1	C113—C114—C121	121.1 (4)
C8—C9—H9	121.1	C115—C114—C121	121.4 (4)
C9—C10—C5	122.5 (4)	C116—C115—C120	117.3 (4)
C9—C10—H10	118.8	C116—C115—C114	119.2 (4)
C5—C10—H10	118.8	C120—C115—C114	123.5 (4)
C12—N11—C16	118.3 (4)	N111—C116—C117	118.0 (4)
C12—N11—Cu1	120.9 (3)	N111—C116—C115	121.5 (4)
C16—N11—Cu1	120.8 (3)	C117—C116—C115	120.5 (4)
N11—C12—C13	124.1 (4)	C118—C117—C116	120.2 (4)
N11—C12—H12	117.9	C118—C117—H117	119.9
C13—C12—H12	117.9	C116—C117—H117	119.9
C14—C13—C12	119.6 (4)	C117—C118—C119	120.4 (4)
C14—C13—H13	120.2	C117—C118—H118	119.8
C12—C13—H13	120.2	C119—C118—H118	119.8
C13—C14—C15	118.4 (4)	C120—C119—C118	120.1 (4)
C13—C14—C21	120.6 (4)	C120—C119—H119	119.9
C15—C14—C21	121.0 (4)	C118—C119—H119	119.9
C20—C15—C16	118.0 (4)	C119—C120—C115	121.4 (4)
C20—C15—C14	123.4 (4)	C119—C120—H120	119.3
C16—C15—C14	118.6 (4)	C115—C120—H120	119.3
N11—C16—C17	119.1 (4)	C114—C121—H12A	109.5
N11—C16—C15	120.9 (4)	C114—C121—H12B	109.5
C17—C16—C15	120.0 (4)	H12A—C121—H12B	109.5
C18—C17—C16	119.9 (4)	C114—C121—H12C	109.5
C18—C17—H17	120.0	H12A—C121—H12C	109.5
C16—C17—H17	120.0	H12B—C121—H12C	109.5
			
O3—Cu1—O1—C1	88.7 (5)	C14—C15—C16—C17	−179.4 (4)
N3—Cu1—O1—C1	11.4 (3)	N11—C16—C17—C18	178.3 (4)
N11—Cu1—O1—C1	−171.9 (3)	C15—C16—C17—C18	−0.8 (6)
O4—Cu1—O1—C1	−78.4 (3)	C16—C17—C18—C19	0.3 (7)
N3—Cu1—O3—C6	6.8 (3)	C17—C18—C19—C20	−0.6 (7)
O1—Cu1—O3—C6	−69.4 (6)	C18—C19—C20—C15	1.2 (7)
N11—Cu1—O3—C6	−168.6 (3)	C16—C15—C20—C19	−1.7 (6)
O4—Cu1—O3—C6	97.3 (3)	C14—C15—C20—C19	179.2 (4)
O103—Cu2—O101—C101	−87.7 (6)	Cu2—O101—C101—O102	−174.3 (3)
N103—Cu2—O101—C101	−11.9 (3)	Cu2—O101—C101—C102	3.4 (4)
N111—Cu2—O101—C101	172.8 (3)	O102—C101—C102—N103	−171.8 (3)
O104—Cu2—O101—C101	78.8 (3)	O101—C101—C102—N103	10.4 (5)
N103—Cu2—O103—C106	−1.9 (3)	C101—C102—N103—C104	165.2 (3)
N111—Cu2—O103—C106	172.3 (3)	C101—C102—N103—Cu2	−19.5 (4)
O101—Cu2—O103—C106	72.8 (6)	O103—Cu2—N103—C104	−0.5 (4)
O104—Cu2—O103—C106	−93.2 (3)	O101—Cu2—N103—C104	−167.7 (4)
Cu1—O1—C1—O2	174.8 (3)	O104—Cu2—N103—C104	104.2 (3)
Cu1—O1—C1—C2	−3.8 (4)	O103—Cu2—N103—C102	−175.4 (3)
O2—C1—C2—N3	172.2 (3)	O101—Cu2—N103—C102	17.4 (3)
O1—C1—C2—N3	−9.0 (5)	O104—Cu2—N103—C102	−70.7 (3)
C1—C2—N3—C4	−161.0 (4)	C102—N103—C104—C105	176.0 (4)
C1—C2—N3—Cu1	17.7 (4)	Cu2—N103—C104—C105	1.3 (6)
O3—Cu1—N3—C4	−4.4 (4)	N103—C104—C105—C110	−178.5 (4)
O1—Cu1—N3—C4	162.6 (4)	N103—C104—C105—C106	−0.1 (6)
O4—Cu1—N3—C4	−109.0 (3)	Cu2—O103—C106—C107	−178.5 (3)
O3—Cu1—N3—C2	177.0 (3)	Cu2—O103—C106—C105	3.4 (6)
O1—Cu1—N3—C2	−16.0 (3)	C110—C105—C106—O103	175.9 (4)
O4—Cu1—N3—C2	72.4 (3)	C104—C105—C106—O103	−2.4 (6)
C2—N3—C4—C5	−179.3 (4)	C110—C105—C106—C107	−2.2 (6)
Cu1—N3—C4—C5	2.2 (6)	C104—C105—C106—C107	179.4 (4)
N3—C4—C5—C10	179.6 (4)	O103—C106—C107—C108	−175.5 (4)
N3—C4—C5—C6	−0.6 (6)	C105—C106—C107—C108	2.7 (6)
Cu1—O3—C6—C7	174.1 (3)	C106—C107—C108—C109	−1.4 (7)
Cu1—O3—C6—C5	−7.3 (6)	C107—C108—C109—C110	−0.6 (7)
C10—C5—C6—O3	−176.9 (4)	C108—C109—C110—C105	1.1 (7)
C4—C5—C6—O3	3.3 (6)	C106—C105—C110—C109	0.4 (6)
C10—C5—C6—C7	1.6 (6)	C104—C105—C110—C109	178.8 (4)
C4—C5—C6—C7	−178.1 (4)	O103—Cu2—N111—C112	85.4 (3)
O3—C6—C7—C8	177.2 (4)	O101—Cu2—N111—C112	−107.6 (3)
C5—C6—C7—C8	−1.5 (6)	O104—Cu2—N111—C112	−19.4 (3)
C6—C7—C8—C9	0.1 (7)	O103—Cu2—N111—C116	−97.9 (3)
C7—C8—C9—C10	1.2 (7)	O101—Cu2—N111—C116	69.1 (3)
C8—C9—C10—C5	−1.0 (7)	O104—Cu2—N111—C116	157.4 (3)
C6—C5—C10—C9	−0.4 (6)	C116—N111—C112—C113	0.8 (6)
C4—C5—C10—C9	179.3 (4)	Cu2—N111—C112—C113	177.7 (3)
O3—Cu1—N11—C12	−86.6 (3)	N111—C112—C113—C114	−0.5 (6)
O1—Cu1—N11—C12	106.6 (3)	C112—C113—C114—C115	0.6 (6)
O4—Cu1—N11—C12	18.3 (3)	C112—C113—C114—C121	−179.6 (4)
O3—Cu1—N11—C16	94.1 (3)	C113—C114—C115—C116	−1.1 (6)
O1—Cu1—N11—C16	−72.7 (3)	C121—C114—C115—C116	179.2 (4)
O4—Cu1—N11—C16	−161.1 (3)	C113—C114—C115—C120	−179.9 (4)
C16—N11—C12—C13	1.8 (6)	C121—C114—C115—C120	0.3 (7)
Cu1—N11—C12—C13	−177.5 (3)	C112—N111—C116—C117	179.7 (4)
N11—C12—C13—C14	0.6 (6)	Cu2—N111—C116—C117	2.8 (5)
C12—C13—C14—C15	−1.9 (6)	C112—N111—C116—C115	−1.3 (6)
C12—C13—C14—C21	179.6 (4)	Cu2—N111—C116—C115	−178.2 (3)
C13—C14—C15—C20	180.0 (4)	C120—C115—C116—N111	−179.6 (4)
C21—C14—C15—C20	−1.5 (6)	C114—C115—C116—N111	1.5 (6)
C13—C14—C15—C16	0.8 (6)	C120—C115—C116—C117	−0.6 (6)
C21—C14—C15—C16	179.3 (4)	C114—C115—C116—C117	−179.6 (4)
C12—N11—C16—C17	178.1 (4)	N111—C116—C117—C118	−179.6 (4)
Cu1—N11—C16—C17	−2.6 (5)	C115—C116—C117—C118	1.4 (6)
C12—N11—C16—C15	−2.9 (6)	C116—C117—C118—C119	−0.9 (6)
Cu1—N11—C16—C15	176.5 (3)	C117—C118—C119—C120	−0.4 (6)
C20—C15—C16—N11	−177.6 (4)	C118—C119—C120—C115	1.1 (6)
C14—C15—C16—N11	1.6 (6)	C116—C115—C120—C119	−0.6 (6)
C20—C15—C16—C17	1.4 (6)	C114—C115—C120—C119	178.3 (4)

### Hydrogen-bond geometry (Å, °)

**Table:**

*D*—H···*A*	*D*—H	H···*A*	*D*···*A*	*D*—H···*A*
O4—H4V···O102^i^	0.879 (19)	1.88 (2)	2.756 (4)	174 (4)
O4—H4W···O2^ii^	0.87 (4)	2.01 (3)	2.825 (4)	155 (4)
O5—H5V···O101^iii^	0.890 (19)	1.99 (2)	2.865 (4)	169 (4)
O6—H6V···O1	0.876 (19)	2.01 (2)	2.867 (4)	165 (4)
O104—H4Y···O2	0.879 (19)	1.90 (2)	2.751 (4)	162 (4)
O104—H4Z···O102^iii^	0.87 (4)	1.98 (2)	2.823 (4)	162 (4)

Symmetry codes: (i) *x*+1, *y*, *z*; (ii) −*x*+1, *y*, −*z*+1/2; (iii) −*x*, *y*, −*z*+1/2.
